# A chlorzoxazone–folic acid combination improves cognitive affective decline in SCA2-58Q mice

**DOI:** 10.1038/s41598-023-39331-y

**Published:** 2023-08-03

**Authors:** Ksenia S. Marinina, Ilya B. Bezprozvanny, Polina A. Egorova

**Affiliations:** 1 Laboratory of Molecular Neurodegeneration, Peter the Great St. Petersburg Polytechnic University, St. Petersburg, Russia; 2 Department of Physiology, University of Texas Southwestern Medical Center, Dallas, TX USA

**Keywords:** Cognitive neuroscience, Neurophysiology, Patch clamp

## Abstract

Spinocerebellar ataxia type 2 (SCA2) is a polyglutamine disorder caused by a pathological expansion of CAG repeats in *ATXN2* gene. SCA2 is accompanied by cerebellar degeneration and progressive motor decline. Cerebellar Purkinje cells (PCs) seem to be primarily affected in this disorder. The majority of the ataxia research is focused on the motor decline observed in ataxic patients and animal models of the disease. However, recent evidence from patients and ataxic mice suggests that SCA2 can also share the symptoms of the cerebellar cognitive affective syndrome. We previously reported that SCA2-58Q PC-specific transgenic mice exhibit anxiolytic behavior, decline in spatial memory, and a depressive-like state. Here we studied the effect of the activation of the small conductance calcium-activated potassium channels (SK channels) by chlorzoxazone (CHZ) combined with the folic acid (FA) on the PC firing and also motor, cognitive and affective symptoms in SCA2-58Q mice. We realized that CHZ-FA combination improved motor and cognitive decline as well as ameliorated mood alterations in SCA2-58Q mice without affecting the firing rate of their cerebellar PCs. Our results support the idea of the combination therapy for both ataxia and non-motor symptoms in ataxic mice without affecting the firing frequency of PCs.

## Introduction

Spinocerebellar ataxia type 2 (SCA2) is an autosomal dominant neurodegenerative disorder caused by a pathological expansion of polyglutamine in the ataxin-2 protein. Cerebellar Purkinje cells (PCs) are primarily affected in this disorder and the progressive cerebellar atrophy accompanies the development of the disease. SCA2 is mostly known for its various motor symptoms including but not limited to ataxia. To this day SCA2 stays incurable, no disease-modifying therapy was invented so far. The SCA2 patients are mostly provided with the supporting therapy and the palliative care. In addition to the motor symptoms, SCA2 patients also suffer from the cognitive decline in the verbal and visuospatial memory, executive functions, attention, learning ability and comprehension ability^1–4^. The Hamilton Depression Scale has also revealed signs of mild depression in SCA2 patients with the moderate symptoms of apathy and avolition and mild symptoms of anhedonia/asociality^3,5^. Thus, the SCA2 patients experience not only motor disabilities, but also exhibit the decline in cognition and the negative mood alterations. Of course, the cognitive and affective decline of SCA2 patients might be explained by the awareness of their uncurable status. However, recent evidence suggests that the cerebellum might be directly involved in the cognitive control and the emotional processing.

Indeed, the neurologist Dr. Jeremy Schmahmann studied the patients with the lesions specific to cerebellum, and defined the cerebellar cognitive affective syndrome (CCAS), also known as Schmahmann’s syndrome^6^. Particularly, these patients demonstrated the difficulties in the executive functions, working and spatial memory; they also suffered from the personality changes and language deficits^6^. Furthermore, the subsequent studies on the patients with different psychiatric disorders revealed the correlation between the cognitive symptoms and/or mood deficits and the cerebellar atrophy. Thus, the cerebellar involvement was reported in patients with the autism spectrum disorder^7^, schizophrenia^8^, bipolar disorder^9^, ADHD^10^, and the major depressive disorder^11^. Moreover, the morphometrical assessments in SCA2 patients also revealed the correlation between the cerebellar atrophy and cognitive disability^12,13^.

It is well known that animal models are generally used to study the fundamental bases of the neurodegenerative diseases, including different types of ataxia. Mice models specifically allow us to study the behavior and treatment tolerance that is bringing us closer to being able to find the new possible therapies to take care of the incurable diseases. Pursuant to this, it is very important for mice models to develop symptoms as similar to the patient’s symptoms as possible. Aside from the motor symptoms, greatly described in many ataxic murine models, the cognitive and affective symptoms were also reported in ataxic mice. Thus, SCA1-154Q knock-in mice and PC-specific SCA1-82Q transgenic mice demonstrated the decline in spatial memory and also in cued fear memory^14^. Two different mouse models of episodic ataxia type 2 (EA2) displayed cognitive impairments in recognition memory and demonstrated social interaction deficiencies^15^. In our previous studies we have observed that SCA2-58Q mice exhibit anxiolytic behavior, decline in spatial memory, and a depressive-like state^16^. Thus, the transgenic mouse models of autosomal dominant cerebellar ataxia (ADCA) can be used for finding targets to utilize combined therapy in ADCA for both ataxia and non-motor symptoms.

As mentioned earlier, cerebellar PCs are primarily affected in SCA2. In our lab we mainly focused our research on the PC electrophysiological activity in the SCA2-58Q transgenic mouse model. We have reported that SCA2 PCs fire much less regularly compared to their WT PC controls in acute cerebellar slices^17–19^, in urethane-anesthetized mice^20^, and also in awake head-fixed SCA2-58Q mice^21^. It is known that PC pacemaking is regulated by the small conductance calcium-activated potassium channels (SK channels)^22^. It was assumed that the activation of SK and BK channels might rescue the impaired electrophysiology of ataxic PCs and, furthermore, improve the motor decline reported in ataxic mice^23^. Indeed, the SK channels modulators recovered the abnormal PC firing and subsequently restored the impaired motor activity in SCA1-82Q transgenic mice^24^, in murine models of EA2^25–27^, in mutant *CACNA1A* mice^28^, in SCA3 transgenic mice^29^, in SCA2-58Q mice^18^, and even in the murine model of Huntington’s disease (HD) YAC128^30^. Furthermore, a human trial with SK activator riluzole had been carried out^31^. A randomized, double-blind, placebo-controlled trial on ataxic patients demonstrated that the exposure to 100 mg/day riluzole slightly improved SARA scores in respondents and didn’t exert any severe side effects^31^. Thus, the SK channel-mediated treatment improved motor decline in murine ataxic models and human patients most likely due to the restoration of normal PC firing. However, activation of SK channels slows down pacemaking of neurons^20,32^, which can cause negative side effects in humans. Therefore, in this study we attempted to find a pharmacological agent which would reverse the reduction of firing frequency in PCs caused by SK channel activation. We assumed that this agent would have both neuroprotective and antidepressant effects to facilitate its usage in the combined therapy in SCA2 for both ataxia and non-motor symptoms. Our choice fell on the folic acid (FA) since it is known to have an antidepressant effect both alone^33^ and in combination with traditional antidepressants^34,35^. It is also known that FA affects the morphology of the cerebellum in rodents^36^, and also protects neurons from excitotoxicity^37,38^.

We have demonstrated previously that the SK channel activator chlorzoxazone (CHZ) improves the motor dysfunction in SCA2-58Q mice^21^, but until now no one had reported the effect of CHZ on the cognition and mood alterations. This information would be very useful for studying the combined therapy in SCAs for both ataxia and non-motor symptoms. Here, we planned to investigate the effect of the SK channel activator CHZ together and separately with the FA on the motor and cognitive functions as well as mood alterations in SCA2-58Q PC-specific transgenic mice.

## Methods

### Mice breeding and genotyping

Transgenic SCA2-58Q mice and their wild type (WT) littermates at the age of 12 months were used in these experiments. SCA2-58Q mice^39^ were kindly provided to us by Dr. Stefan Pulst (University of Utah, Salt Lake City, Utah, USA) and were crossed to the FVB background strain. The breeding and genotyping of these mice were previously described^20,21,40^. Briefly, hemizygous male SCA2-58Q mice were crossed with the WT female mice to generate mixed litters. The genotyping was done via PCR for *ATXN2* transgene as previously described^17,18,20^. The volume of one PCR sample was 25 μl. The PCR mix per one sample contained: 2.5 μl 10 × buffer for Taq polymerase, 0.5 μl 10 mM dNTP, 1.5 μl 25 mM MgCl_2_, 0.125 μl 20 μM primers (forward and reverse), 0.25 μl Taq polymerase, 2 μl DNA, and 18 μl dH2O. The sequence of the forward primer is: 5′-GCGAACACAAAGAGAAGGACCTGGA-3′. The sequence of the reverse primer is: 5′-GCCCTTGCTTCCCGTTTTAA-3′. The PCR product has 232 bp. Mice were housed in groups of two to six in one cage in the vivarium. The temperature was kept 22–24 °C including 12 daylight hours. The animals had access to standard food and water ad libitum. All procedures were approved by the Bioethics Committee of the Peter the Great St. Petersburg Polytechnic University at St. Petersburg, Russia and followed the principles of European convention (Strasbourg, 1986) and the Declaration of International medical association about humane treatment of animals (Helsinki, 1996). All methods were carried out in accordance with relevant guidelines and regulations. The study was carried out in compliance with the ARRIVE guidelines.

### Cerebellar slice recordings

Recordings of spontaneous PC activity from WT and SCA2-58Q mice at 12 months of age were performed as previously described^17–19^. Briefly, the mice were anesthetized with 2000 mg/kg urethane and transcardially perfused with ice-cold aCSF containing (mM) 85 NaCl, 24 NaHCO_3_, 25 glucose, 2.5 KCl, 0.5 CaCl_2_, 4 MgCl_2_, 1 NaH_2_PO_4_, 75 sucrose. Solutions were equilibrated with carbogen (95% O_2_/5% CO_2_). Next, the cerebellum was dissected and 300 μm thick sagittal slices were cut with a VT1200S vibratome (Leica). Slices were recovering in aCSF containing (in mM) 119 NaCl, 26 NaHCO_3_, 11 glucose, 2.5 KCl, 2.5 CaCl_2_, 1.3 MgCl_2_, 1 NaH_2_PO_4_ at 35 °C for 30–40 min and then transferred to the room temperature before recordings started. The external bath solution used for the recording was the same as the recovery aCSF, but also contained 100 μM picrotoxin (PTX) and 10 μM 6,7-dinitroquinoxaline-2,3-dione (DNQX), equilibrated with carbogen. All recordings were made within 6–7 h after cerebellum was dissected. The recording chamber was heated to 35 °C using TC-324C automatic temperature controller (Warner Instruments, Hamden, CT). Loose-patch recordings were made as described previously^17–19^ to evaluate the spontaneous activity of cerebellar PCs. Briefly, 1.5–3 MΩ glass pipettes were filled with the internal solution containing 140 mM NaCl buffered with 10 mM HEPES pH 7.3 and held at 0 mV. A loose patch (< 100 MΩ) configuration was established at the PC soma at the axon hillock area. Spontaneous action potential currents were recorded for 5 min from each cell using Axon Multiclamp 700B amplifier (Molecular Devices, Sunnyvale, CA). The recordings were analyzed for tonic or burst firing as we previously described^17–20^. Cells were characterized as firing tonically if they fired repetitive nonhalting spike trains for 5 min. A cell was characterized as bursting if it had more than 5% of the interspike intervals that fell outside of 3 SD from the mean of all interspike intervals in that cell. The analysis of PC firing was performed using Clampfit 10.2 (Molecular Devices). When studying the effect of the drugs, the 30 μM chlorzoxazone (CHZ), 50 μM folic acid (FA), or both (CHZ-FA) were added to the recording solution.

### Drug delivery in mice

Mice from each litter were genotyped, weight-matched, and divided into four WT and four transgenic SCA2-58Q groups, with each group containing 6–10 mice. Experimental animals were given intraperitoneal (i. p.) injections with diluted in 5% DMSO in PBS 30 mg/kg CHZ or 30 mg/kg FA or both (30 mg/kg CHZ + 30 mg/kg FA) 5 days a week with a 2 days break during 6 weeks period. The control groups of animals were i. p. injected with a vehicle following the same scheme. The injections were done to mice from 10.5 to 12 months of age.

**Figure 1:**
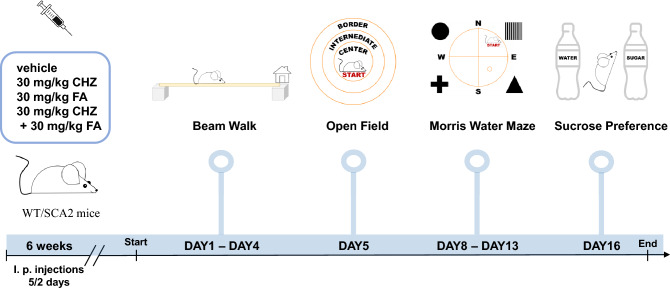
A graphical time scheme of the CHZ-FA trial in SCA2-58Q mice. WT and SCA2-58Q mice at the age of 10.5 months were i. p. injected with vehicle, CHZ, FA, or CHZ-FA combination during 6 weeks. Following this, the beam walk assay was used to evaluate the motor activity, the open field test was used to assess the level of anxiety, the Morris water maze was used to describe the spatial learning, and the sucrose preference test was used to measure the level of depression.

The distribution of mice by sex was the following within the groups: 7 females + 1 male WT Ctrl, 6 females + 2 males WT CHZ, 7 females WT FA, and 7 females + 1 male WT CHZ-FA; 8 females + 2 males SCA2 Ctrl, 4 females + 3 males SCA2 CHZ, 3 females + 4 males SCA2 FA, and 3 females + 3 males SCA2 CHZ-FA. A graphical time scale related to the design of the experiments, including treatment and different behavioral tests, is shown on the Fig. 1.

### Motor activity assessment

The assessment of motor decline in SCA2-58Q mice was carried out as previously described^21,30^. The beam walk test was conducted via a home-made experimental setup including a beam of 1.5 m suspended 0.5 m above the ground. The 18 mm, 12 mm, and 8 mm round wood beams were used in our studies. During the beam walk test, the experimental animals were trained on beams for 3 consecutive days (three training sessions per day for each beam) to traverse the beam to the enclosed box. Once the stable baseline of performance was obtained, the mice were tested in three consecutive trials on 18 mm, 12 mm, and 8 mm round wood beams, in each case progressing from the widest to the narrowest beam. The latency to traverse the middle 1 m of each beam and the number of times the hind paws slipped off each beam were recorded for each trial. For each experimental group, the average value of the three trials for each beam were used in the analysis.

### Behavior tests

#### Open field

The open field arena consisted of opaque, plexiglass chamber with a diameter of 62.4 cm subdivided into a center (with a diameter of 28.4 cm), intermediate and border (8.5 cm from chamber wall) regions, which were brightly illuminated with 950 lx above the arena^15,41^. During the test mice were placed into the center of the open field and the following parameters were video tracked for 10 min: time spent in the center, border, and the intermediate zones, and total distance moved. The apparatus was cleaned between subjects with 70% ethanol. Each mouse underwent 1 trial. The results were analyzed via the Any-maze software.

#### Morris water maze

The Morris water maze (MWM) was used to evaluate spatial learning and memory. A circular white pool (150 cm diameter, 66 cm high, and 29 cm deep) was filled with opaque water (21–23 °C). A circular platform (10 cm diameter, 27.5 cm high) was placed in the pool and was submerged ∼ 1.5 cm below the surface of the opaque water. To find the hidden platform, the experimental animals were supposed to navigate via four black and white cues positioned at the four intercardinal directions 10 cm above the water surface. The trials were recorded via VS 1304-1 video system with Gigabit Ethernet software. Mice were trained for 4 consecutive days with 4 trials per day. Between each trial mice were having a break for at least 15 min to dry out and rest. During each trial mice had 90 s to find the hidden platform. If mice were not able to find the platform within 90 s, they were guided to the platform by the observer and allowed to rest on the platform for at least 15 s. The Any-maze software was used to evaluate the number of successful trials and the average latency to find the platform during the training sessions. On the last (fifth) day of the test, one 90 s trial with no platform was performed for each mouse. Time spent in SE quadrant (%), number of intersections of the platform position, and total distance swam on the fifth day were analyzed also via the Any-maze software.

#### Sucrose preference test

The sucrose preference test (SPT) was performed to assess the anhedonic behavior in SCA2-58Q mice. Briefly, mice were given a choice between two bottles, one with 2% sucrose solution, and another one with water. Mice were habituated to the bottles for at least 2 days before the experiment. The experiment was carried out for 8 h. During the experiment no food was allowed to the experimental animal. The position of the bottles was changed after 4 h to avoid the possibility of bottle location preference by the mouse under test. The consumption of water and sucrose solution was assessed by evaluation of the liquid volume in the bottles. The preference for sucrose expressed in percent was calculated as a ratio of the intake of the sucrose solution to the total fluid intake, multiplied by a hundred.

### Statistical analysis

Sample sizes were calculated using power analyses based on the standard deviations from our previous studies, significance level of 5%, and power of 90%. Statistical tests were performed with Origin software. Data were analyzed using one-way ANOVA/Bonferroni post-test. Data were presented as bar graphs with SD bars and overlaid dot plots and were plotted in GraphPad Prism. Significance for comparisons: *p < 0.05; **p < 0.01; ****p < 0.0001. The number of mice for every experiment is reported in the figures.

## Results

### Chlorzoxazone-folic acid recovers the firing precision in SCA2-58Q PCs without affecting their firing rate

**Figure 2:**
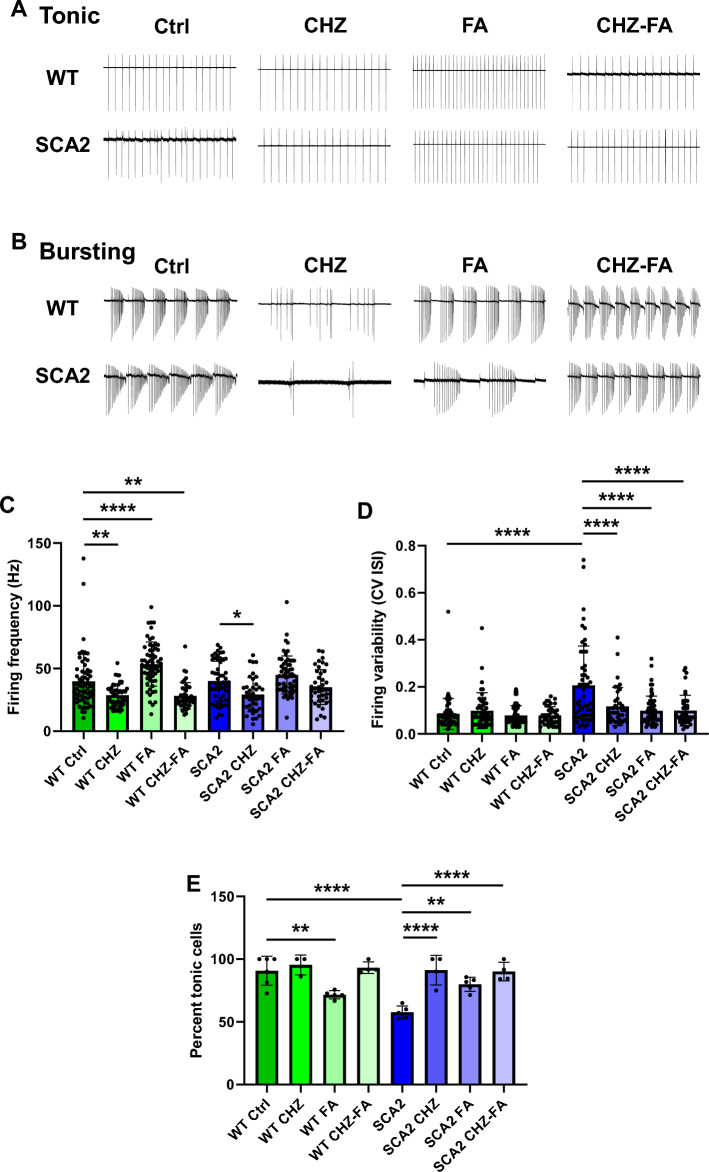
Chlorzoxazone-folic acid improves PC firing precision in SCA2-58Q mice. (**A**) Examples of tonic PC activity in 12-month-old WT and SCA2-58Q mice in control groups and in the presence of CHZ, FA, and CHZ-FA combination. Recording traces 600 ms duration are shown. (**B**) Examples of bursting PC activity in 12-month-old WT and SCA2-58Q mice in control groups and in the presence of CHZ, FA, and CHZ-FA. Recording traces 600 ms duration are shown. (**C**) The average firing frequencies for PCs in WT Ctrl, WT CHZ, WT FA, and WT CHZ-FA groups; and for PCs in SCA2 Ctrl, SCA2 CHZ, SCA2 FA, and SCA2 CHZ-FA groups are shown as bar graphs with SD bars and overlaid dot plots. Number of tonic cells (n) and the number of mice (m): n = 62 WT Ctrl PCs, m = 6 WT Ctrl mice; n = 47 WT CHZ PCs, m = 3 WT CHZ mice; n = 60 WT FA PCs, m = 5 WT FA mice; n = 41 WT CHZ-FA PCs, m = 4 WT CHZ-FA mice; n = 54 SCA2 Ctrl PCs, m = 5 SCA2 Ctrl mice; n = 39 SCA2 CHZ PCs, m = 4 SCA2 CHZ mice; n = 59 SCA2 FA PCs, m = 5 SCA2 FA mice; n = 42 SCA2 CHZ-FA PCs, m = 4 SCA2 CHZ-FA mice. (**D**) The CV ISI was analyzed as a measure of the firing precision for the groups described in (**C**). (**E**) The proportion of PCs firing tonically was calculated as a percentage of total active cells and plotted as means ± SD. Number of total active cells: n = 71 WT Ctrl PCs; n = 51 WT CHZ PCs; n = 83 WT FA PCs; n = 45 WT CHZ-FA PCs; n = 95 SCA2 Ctrl PCs; n = 45 SCA2 CHZ PCs; n = 73 SCA2 FA PCs; n = 48 SCA2 CHZ-FA PCs. The number of mice tested per group is indicated in (**C**). *p < 0.05; **p < 0.01; ****p < 0.0001 (one-way ANOVA/Bonferroni post-test).

In our previous research^17,42^ we have demonstrated the correlation between the impairments in the spontaneous activity of SCA2-58Q Purkinje cells (PCs) and PC cell death. Thus, the dark cell degeneration quantification, which was used as a measure of the health state of PCs, has demonstrated that the amount of the severely degenerated PCs was the same as the portion of bursting PCs in patch-clamp experiments^17^. Therefore, in this study the assessment of the PC electrophysiological status was used as a measure of the neuronal functionality. In our experiments we studied the electrophysiological activity of cerebellar PCs in acute cerebellar slices via the loose patch configuration of the patch clamp method. In the current trial we used the following experimental groups for cerebellar slice recordings: WT and SCA2 control (Ctrl), WT and SCA2 PCs in the presence of 30 μM chlorzoxazone (CHZ), WT and SCA2 PCs in the presence of 50 μM folic acid (FA), and finally WT and SCA2 PCs in the presence of both CHZ and FA (30 μM CHZ + 50 μM FA). Drugs were added to the recording solution. We observed both tonic (Fig. 2A) and bursting (Fig. 2B) PCs in all the groups tested, although the firing frequency was considerably lower inside the bursts in both CHZ groups (Fig. 2B). The average firing frequency was equal in WT Ctrl and SCA2 Ctrl groups (Fig. 2C). Thus, the average firing frequency for the WT Ctrl mice was 39.72 ± 2.72 Hz (n = 62 cells, m = 6 mice) and for SCA2 Ctrl mice it was 39.99 ± 2.15 Hz (n = 54 cells, m = 5 mice, *p* = 0.94). The addition of 30 μM CHZ to the recording solution significantly reduced the firing frequency in both WT and SCA2 PCs (Fig. 2C). Thus, the average firing frequency for the WT CHZ PCs was 28.64 ± 1.29 Hz (n = 47 cells, m = 3 mice, **p < 0.01 vs WT Ctrl group) and for SCA2 CHZ PCs it was 29.25 ± 2.20 Hz (n = 39 cells, m = 4 mice, **p < 0.01 vs WT Ctrl group). On the contrary, the addition of the 50 μM FA to the recording solution increased the firing frequency in both WT and SCA2 PCs, although in SCA2 FA group it did not reach the statistically significant difference (Fig. 2C). Thus, the average firing frequency for the WT FA PCs was 53.49 ± 2.26 Hz (n = 60 cells, m = 5 mice, ****p < 0.0001 vs WT Ctrl group) and for SCA2 FA PCs it was 44.83 ± 1.97 Hz (n = 59 cells, m = 5 mice, *p* = 0.13 vs WT Ctrl group). Finally, when both 30 μM CHZ and 50 μM FA were added to the recording solution, the average firing frequency was significantly reduced in WT PCs, but it did not change in the SCA2 CHZ-FA group (Fig. 2C). Thus, the average firing frequency for the WT CHZ-FA PCs was 28.14 ± 1.64 Hz (n = 41 cells, m = 4 mice, **p < 0.01 vs WT Ctrl group) and for SCA2 CHZ-FA PCs it was 35.12 ± 2.15 Hz (n = 42 cells, m = 4 mice, *p* = 0.22 vs WT Ctrl group).

In this study we also observed that both CHZ and FA improved the firing precision of the SCA2-58Q PCs (Fig. 2D, E). Here, the firing variability was assessed as the coefficient of variation (CV) of the interspike intervals (ISI) calculated as a ratio of the standard deviation to the ISI average mean. The CV ISI was significantly increased in the SCA2 Ctrl group compared to their WT Ctrl littermates (Fig. 2D). Thus, the average CV ISI for the WT Ctrl mice was 0.08 ± 0.01 (n = 62 cells, m = 6 mice) and for SCA2 Ctrl mice it was 0.21 ± 0.02 (n = 54 cells, m = 5 mice, **** *p* < 0.0001). Furthermore, the percentage of tonic cells was significantly lower in SCA2 Ctrl mice compared to their WT Ctrl littermates (Fig. 2E). Thus, 90.70 ± 4.72% PCs were firing tonically in WT Ctrl mice (n = 71 cells, m = 6 mice), whereas only 57.62 ± 2.28% tonic PCs were observed in SCA2 Ctrl group (n = 95 cells, m = 5 mice). The addition of 30 μM CHZ to the recording solution did not affect the firing precision of WT PCs, but increased the number of tonic cells in SCA2 CHZ group (Fig. 2D, E). Thus, the average CV ISI for the WT CHZ PCs was 0.10 ± 0.01 (n = 47 cells, m = 3 mice, *p* = 0.33 vs WT Ctrl group) and for SCA2 CHZ PCs it was 0.12 ± 0.01 (n = 39 cells, m = 4 mice, *p < 0.05 vs WT Ctrl group). The percentage of tonic cells was 95.40 ± 4.60% for the WT CHZ group (n = 51 cells, m = 3 mice, *p* = 0.55 vs WT Ctrl group) and for SCA2 CHZ PCs it was 91.25 ± 5.91% (n = 45 cells, m = 4 mice, *p* = 0.94 vs WT Ctrl group). The addition of the 50 μM FA to the recording solution decreased the number of tonic cells in WT mice and improved the firing precision in SCA2 mice (Fig. 2D, E). Thus, the average CV ISI for the WT FA PCs was 0.08 ± 0.01 (n = 60 cells, m = 5 mice, *p* = 0.54 vs WT Ctrl group) and for SCA2 FA PCs it was 0.10 ± 0.01 (n = 59 cells, m = 5 mice, *p* = 0.25 vs WT Ctrl group). The percentage of tonic cells was 71.62 ± 1.48% for the WT FA group (n = 83 cells, m = 5 mice, **p < 0.01 vs WT Ctrl group) and for SCA2 FA PCs it was 79.91 ± 2.53% (n = 73 cells, m = 5 mice, *p* = 0.09 vs WT Ctrl group). The CHZ-FA combination did not affect the firing precision of WT PCs, but improved it in SCA2 PCs (Fig. 2D, E). Thus, the average CV ISI for the WT CHZ-FA PCs was 0.08 ± 0.01 (n = 41 cells, m = 4 mice, *p* = 0.55 vs WT Ctrl group) and for SCA2 CHZ-FA PCs it was 0.10 ± 0.01 (n = 42 cells, m = 4 mice, *p* = 0.29 vs WT Ctrl group). The percentage of tonic cells was 93.20 ± 2.34% for the WT CHZ-FA group (n = 45 cells, m = 4 mice, *p* = 0.70 vs WT Ctrl group) and for SCA2 CHZ-FA PCs it was 90.12 ± 3.71% (n = 48 cells, m = 4 mice, *p* = 0.93 vs WT Ctrl group).

Thus, similar to our previous results^17,19^, here we also demonstrated that SCA2-58Q PCs fire less tonically and exhibit more bursting patterns in 12-month-old SCA2 mice compared to their WT controls, and the precision of the tonic PCs is also impaired in SCA2 mice. The application of CHZ significantly reduces the firing frequency in both WT and SCA2 PCs, on the contrary, FA increases the firing frequency in cerebellar PCs. Both drugs used improve the firing precision of SCA2 PCs. However, the CHZ-FA combination restores the firing precision in SCA2-58Q PCs without affecting the firing rate.

### CHZ-FA combination, but not FA, recovers the impaired motor activity in SCA2-58Q mice

**Figure 3:**
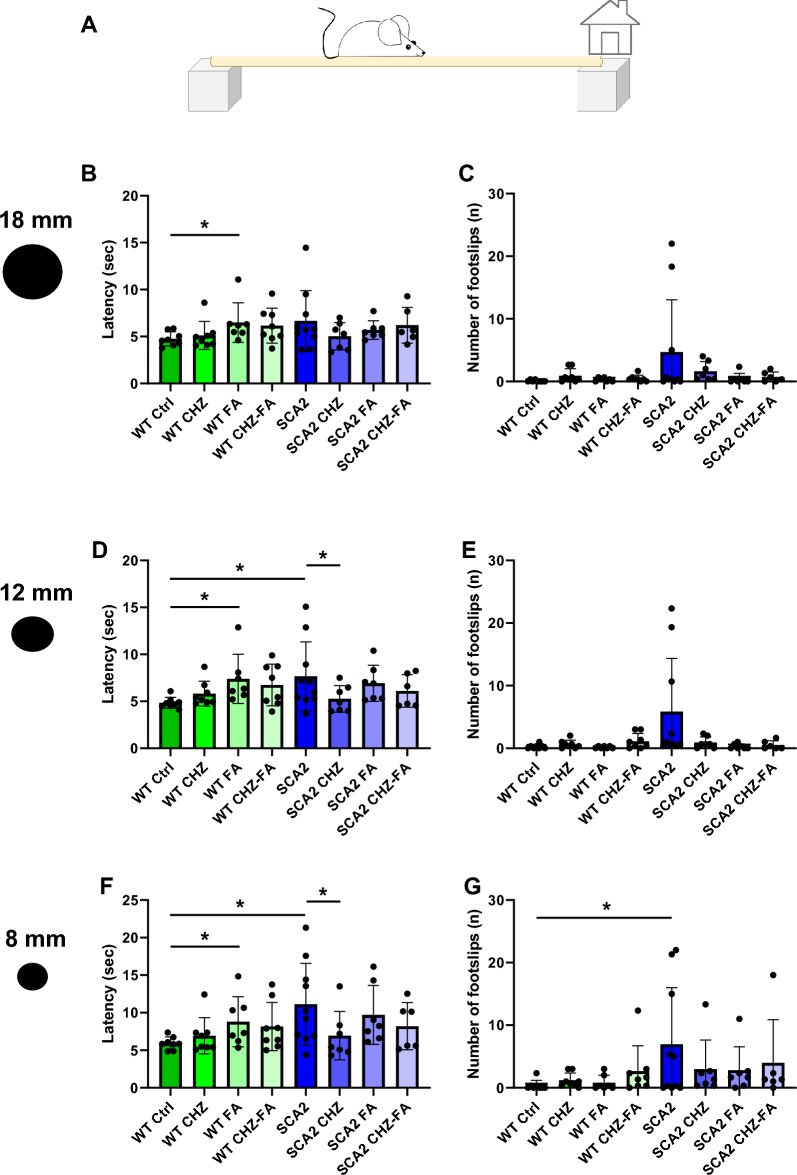
Chlorzoxazone-folic acid combination improves motor decline in SCA2-58Q mice. (**A**) Scheme of the beam walk assay. The average mean latency as the mice traverse the 1 m length of the round 18 mm (**B**), 12 mm (**D**), and 8 mm (**F**) beam is plotted for the 12-month-old WT and SCA2 mice in control groups and after the long-term injections with CHZ, FA, and CHZ-FA combination as bar graphs with SD bars and overlaid dot plots. The average number of foot slips as the mice traverse the 1 m length of the 18 mm (**C**), 12 mm (**E**), and 8 mm (**G**) beam is plotted for the 12-month-old WT and SCA2-58Q mice in control, CHZ, FA, and CHZ-FA groups as means ± SD. The number of mice tested per group (m): m = 8 WT Ctrl mice, m = 8 WT CHZ mice, m = 7 WT FA mice, m = 8 WT CHZ-FA mice; m = 10 SCA2 Ctrl mice, m = 7 SCA2 CHZ mice, m = 7 SCA2 FA mice, m = 6 SCA2 CHZ-FA mice. *p < 0.05 (one-way ANOVA/Bonferroni post-test).

In our previous experiments we have demonstrated that long-term injections of CHZ improved the impaired motor coordination in SCA2-58Q mice^21^. Similar to our current results, we previously reported that CHZ alleviated the precision of the PC activity in awake head-fixed SCA2-58Q mice^21^. Here we also observed that CHZ improved the firing precision in SCA2-58Q PCs. However, the FA exhibited ambiguous effect—while improving firing precision in SCA2 PCs (Fig. 2D), in WT mice the percentage of tonic cells decreased in WT FA group (Fig. 2E). We further decided to study the effect of FA and CHZ-FA combination on the motor activity of the experimental mice (Fig. 3).

The beam walk test was used to assess the motor performance of the eight studied experimental groups at 12 months of age. The average body weight was equal between all test groups (data not shown). The 18 mm, 12 mm, and 8 mm round wood beams were used in this test. To assess the motor coordination of the experimental animals, parameters such as latency and the number of footslips were analyzed using each beam^18,21,30^. The beam walk assay demonstrated that SCA2 Ctrl mice required much more time to traverse the 12 mm (Fig. 3D) and 8 mm (Fig. 3F) beams and their rear paws slipped off the 8 mm (Fig. 3G) beam much more frequently compared to their WT Ctrl littermates. The long-term i. p. injections of 30 mg/kg CHZ recovered the beam walk performance of SCA2 mice (Fig. 3D, F, G). CHZ injections did not affect the motor coordination of WT mice (Fig. 3). The long-term i. p. injections of 30 mg/kg FA resulted in longer time to traverse all test beams without affecting the number of foot slips in WT mice (*p < 0.05) and in SCA2 mice compared to WT Ctrl group (*p < 0.05; Fig. 3). However, the long-term i. p. injections of the 30 mg/kg CHZ + 30 mg/kg FA combination alleviated the motor decline observed in SCA2-58Q ataxic mice as these mice required the same time to traverse the test beams and their paws were slipping off with the same frequency compared to their WT CHZ-FA littermates (Fig. 3). Moreover, the motor performance of SCA2 CHZ-FA mice was also statistically undistinguishable from WT Ctrl mice (Fig. 3). Moreover, the CHZ-FA combination did not affect the WT mice performance (Fig. 3).

### Chlorzoxazone and folic acid reduce the anxiolytic behavior observed in SCA2-58Q mice

**Figure 4:**
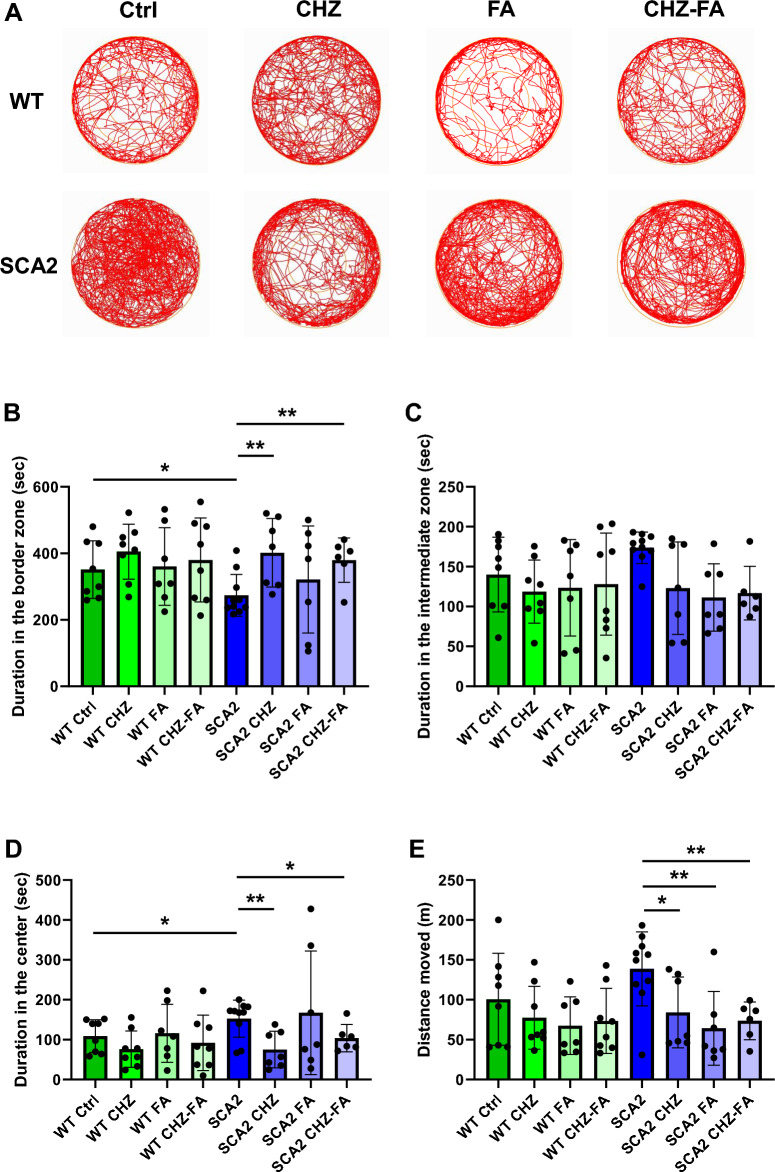
Chlorzoxazone-folic acid combination reduces the anxiolytic behavior in SCA2-58Q mice in the open field test. (**A**) During the open field test, mice were placed in the center of the arena at the start of the test and their behavior was recorded for 10 min. The tracks from 12-month-old WT and SCA2-58Q mice in control groups and after the long-term injections with CHZ, FA, and CHZ-FA combination are shown. (**B**) Average time spent in the border zone by WT and SCA2 mice in control, CHZ, FA, and CHZ-FA groups is shown as bar graphs with SD bars and overlaid dot plots. (**C**) Average time spent in the intermediate zone by WT and SCA2 mice in control, CHZ, FA, and CHZ-FA groups. (**D**) Average time spent in the center of the open field arena by WT and SCA2 mice in control, CHZ, FA, and CHZ-FA groups. (**E**) Total distance moved for WT and SCA2-58Q mice in control, CHZ, FA, and CHZ-FA groups. The number of mice tested per group (m): m = 8 WT Ctrl mice, m = 8 WT CHZ mice, m = 7 WT FA mice, m = 8 WT CHZ-FA mice; m = 10 SCA2 Ctrl mice, m = 7 SCA2 CHZ mice, m = 7 SCA2 FA mice, m = 6 SCA2 CHZ-FA mice. *p < 0.05; **p < 0.01 (one-way ANOVA/Bonferroni post-test).

Our previous results have demonstrated that SCA2-58Q mice exhibit anxiolytic behavior in the open field test^16^. We assume that this anxiolytic behavior, observed in PC-specific transgenic ataxic mice by us^16^ and others^43^ can be explained by the same mechanisms as ADHD symptoms^44^. Here we decided to study the effect of CHZ and/or FA on the anxiety in SCA2 mice and their WT littermates (Fig. 4). We generally observed that the treatments with CHZ, FA, and CHZ-FA combination resulted in the behavioral changes in SCA2 mice as they spent more time in the border zone and less time in the center of the brightly illuminated arena compared to their SCA2 Ctrl littermates (Fig. 4A). Thus, during the open field test, the SCA2 Ctrl mice spent much less time in the border zone (Fig. 4B) and much more time in the central area (Fig. 4D) compared to their WT Ctrl littermates. Noteworthy, the observed anxiolytic phenotype cannot be attributed to ataxia since the average total distance moved was similar in the control groups of SCA2 and WT mice (Fig. 4E), and the median related to the total distance was even greater in SCA2 mice compared to their WT littermates, although it did not reach the statistically significant difference (*p* = 0.14). The applied treatments with CHZ, FA, and CHZ-FA combination generally did not affect the anxious behavior of WT mice (Fig. 4). The long-term i. p. injections of 30 mg/kg CHZ significantly reduced the anxiolytic behavior in SCA2 mice (Fig. 4B, D). Thus, SCA2 CHZ mice spent much more time in the border zone (**p < 0.01 vs SCA2 Ctrl) and much less time in the center (**p < 0.01 vs SCA2 Ctrl) compared to their SCA2 Ctrl littermates. The SCA2 FA mice demonstrated the higher variability of the performances in the open field test (Fig. 4). Thus, there was no significant difference in time spent by SCA2 FA mice in the border zone compared to both WT Ctrl (*p* = 0.66) and SCA2 Ctrl (*p* = 0.41) groups (Fig. 4B). Similar results were observed in time spent by SCA2 FA mice in the center of the arena (*p* = 0.32 vs WT Ctrl and *p* = 0.78 vs SCA2 Ctrl). However, the CHZ-FA combination significantly reduced the anxiolytic behavior in SCA2 mice (Fig. 4B, D) as they spent much more time in the border zone (**p < 0.01 vs SCA2 Ctrl) and much less time in the center (*p < 0.05 vs SCA2 Ctrl) compared to their SCA2 Ctrl littermates. Noteworthy, the application of CHZ, FA, and CHZ-FA significantly decreased the average distance travelled by SCA2 mice in the OF test, and the reduced locomotor activity was similar to the groups of WT mice (Fig. 4E).

### Chlorzoxazone–folic acid has a tendency to recover the learning strategies in SCA2-58Q mice

**Figure 5:**
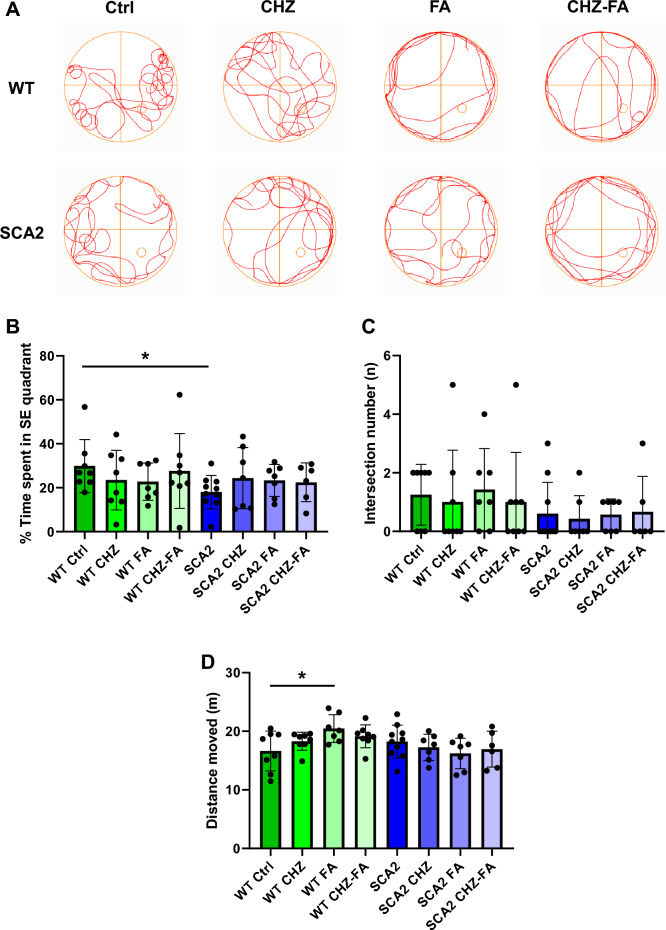
Chlorzoxazone-folic acid have a tendency to improve the learning strategies in SCA2-58Q mice during the Morris water maze task. (**A**) During the Morris water maze test, mice were trained for four consecutive days to find a hidden platform and on the fifth day their behavior was recorded for 90 s with no platform. The tracks from 12-month-old WT and SCA2-58Q mice in control groups and after the long-term injections with CHZ, FA, and CHZ-FA combination are shown on the fifth day. (**B**) Time spent in SE quadrant (%) and (**C**) number of intersections of the platform position for WT and SCA2 mice in control, CHZ, FA, and CHZ-FA groups were plotted on the fifth test day with no platform presented. (**D**) Total distance moved for WT and SCA2-58Q mice in control, CHZ, FA, and CHZ-FA groups on the fifth day. The number of mice tested per group (m): m = 8 WT Ctrl mice, m = 8 WT CHZ mice, m = 7 WT FA mice, m = 8 WT CHZ-FA mice; m = 10 SCA2 Ctrl mice, m = 7 SCA2 CHZ mice, m = 7 SCA2 FA mice, m = 6 SCA2 CHZ-FA mice. *p < 0.05 (one-way ANOVA/Bonferroni post-test).

We have previously demonstrated that SCA2-58Q mice suffer from a cognitive decline in the spatial memory that was observed in the Morris water maze (MWM) test^16^. Here we decided to study the effect of CHZ and/or FA on the spatial memory in SCA2 mice and their WT littermates in the MWM test (Fig. 5). During the MWM test, mice were trained for four consecutive days to find the platform hidden under the opaque water. On the fifth day, mice behavior was recorder during 90 s with no platform. Here we generally observed the improvements in the learning strategies in SCA2 mice after the long-term i. p. injections of 30 mg/kg CHZ, 30 mg/kg FA, and the CHZ-FA combination (Fig. 5A). Thus, on the fifth day, SCA2 Ctrl mice spent much less time in the SE quadrant (where the platform used to be) compared to their WT Ctrl littermates (*p < 0.05), while demonstrating equal number of intersections of the platform site (*p* = 0.21; Fig. 5C). The long-term i. p. injections of CHZ, FA, and the CHZ-FA combination improved the % time spent in SE quadrant (Fig. 5B), although this improvement was due to the increase in the variability of the performances, since there was no statistically significant difference between the experimental SCA2 groups and their SCA2 control littermates (SCA2 CHZ vs SCA2 Ctrl: *p* = 0.24; SCA2 FA vs SCA2 Ctrl: *p* = 0.17; SCA2 CHZ-FA vs SCA2 Ctrl: *p* = 0.31). Interestingly, the long-term FA injections increased the locomotor activity in WT, but not SCA2 mice, as WT FA mice moved longer distances during the fifth day of the MWM task (*p < 0.05; Fig. 5D). Otherwise, treatment with CHZ, FA, and CHZ-FA combination did not affect the performance of WT mice during the MWM task (Fig. 5).

**Figure 6:**
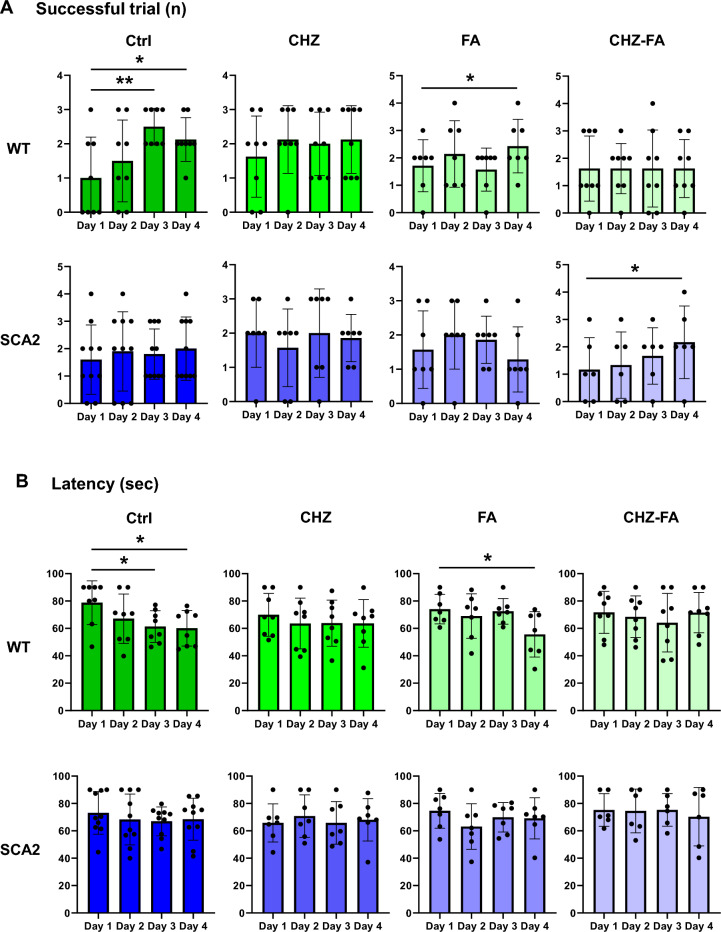
Chlorzoxazone-folic acid have a tendency to improve the spatial learning during training days in the MWM. (**A**) The number of successful trials and (**B**) the average latency to find the hidden platform during four consecutive days of the training sessions in the MWM test for WT and SCA2 mice in control, CHZ, FA, and CHZ-FA groups were plotted as bar graphs with SD bars and overlaid dot plots. The number of mice tested per group (m): m = 8 WT Ctrl mice, m = 8 WT CHZ mice, m = 7 WT FA mice, m = 8 WT CHZ-FA mice; m = 10 SCA2 Ctrl mice, m = 7 SCA2 CHZ mice, m = 7 SCA2 FA mice, m = 6 SCA2 CHZ-FA mice. *p < 0.05; **p < 0.01 (one-way ANOVA/Bonferroni post-test).

The WT Ctrl mice demonstrated the spatial learning in the MWM task as we can see from the values of the latency (Fig. 6B) and the number of successful trials (Fig. 6A) during the four consecutive training days. The WT FA mice also succeeded in this task (Fig. 6). The WT CHZ and WT CHZ-FA mice did not demonstrate the statistically significant success in spatial learning during the training days in MWM task (Fig. 6A, B). The SCA2 Ctrl mice also failed to learn the platform position during the training days in MWM test (Fig. 6A, B). The SCA2 CHZ-FA group exhibited the statistically significant increase in the number of successful trials on the fourth day of the MWM task (Fig. 6A). Although the other compounds tested did not improve the scores of the SCA2 mice during the training days (Fig. 6), their performance had no statistical difference on the fifth testing day compared to their WT Ctrl littermates (Fig. 5B). As we can see from the graphs (Fig. 5B), some of the SCA2 mice under treatment conditions performed much better compared to their SCA2 Ctrl littermates, but some of them didn’t. The possible explanation of this diversity might be due to the retinal damage observed in FVB mice^45^. In our previous research we have demonstrated that SCA2-58Q mice exhibited no alterations in the fear and recognition memory^16^.

### Chlorzoxazone and folic acid ameliorate the depressive-like behavior in SCA2-58Q mice

**Figure 7:**
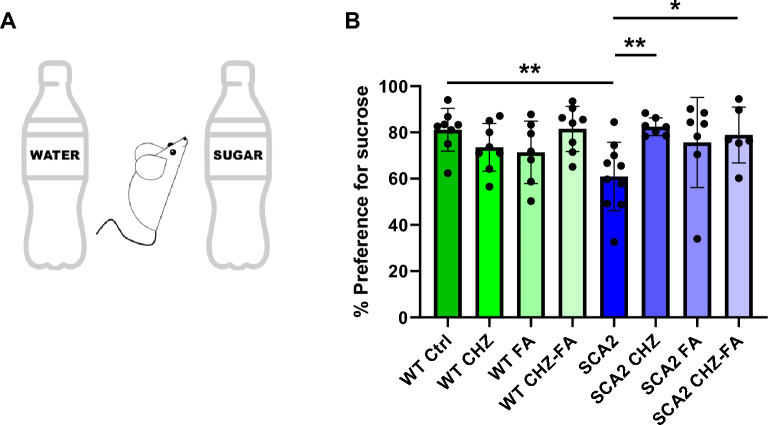
Chlorzoxazone-folic acid ameliorates depressive-like behavior in SCA2-58Q mice. (**A**) During the sucrose preference test mice were given a choice between two bottles, one with 2% sucrose solution, and another one with water. (**B**) Average preference for sucrose solution (%) is shown as mean ± SD for each mice group: WT and SCA2-58Q mice in control groups and after the long-term injections with CHZ, FA, and CHZ-FA combination. The number of mice tested per group (m): m = 8 WT Ctrl mice, m = 8 WT CHZ mice, m = 7 WT FA mice, m = 8 WT CHZ-FA mice; m = 10 SCA2 Ctrl mice, m = 7 SCA2 CHZ mice, m = 7 SCA2 FA mice, m = 6 SCA2 CHZ-FA mice. *p < 0.05; **p < 0.01 (one-way ANOVA/Bonferroni post-test).

Our previous results have demonstrated that SCA2-58Q mice have signs of depression and anhedonia which were observed in the forced swimming test and sucrose preference test (SPT)^16^. Here we decided to study the effect CHZ and/or FA on the mood alterations in SCA2 mice and their WT littermates in the SPT (Fig. 7). During this test, mice were given a choice between two equal bottles of the same volume (80 ml) filled with 50 ml of water or the 2% sucrose solution (Fig. 7A). The test continued for 8 h, in the middle of the test the position of the bottles was interchanged. According to the previous results^16^, here the SCA2 mice have also demonstrated much less preference for sucrose (**p < 0.01) compared to their WT littermates during this task (Fig. 7B). The applied treatments with CHZ, FA, and CHZ-FA combination generally did not affect the sugar consumption of WT mice (Fig. 7B). The long-term i. p. injections of 30 mg/kg CHZ significantly increased the preference for sucrose in SCA2 mice (Fig. 7B). Thus, SCA2 CHZ mice consumed much more sugar compared to their SCA2 Ctrl littermates (**p < 0.01). The SCA2 FA mice also demonstrated the higher preference for sucrose in SPT compared to their SCA2 controls, although this difference has not reached the statistical significance (*p* = 0.10). However, the CHZ-FA combination significantly increased the sugar consumption in SCA2 mice (Fig. 7B) compared to their SCA2 Ctrl littermates (*p < 0.05).

## Discussion

The SCA2-58Q mice used in this study are characterized by a progressive motor dysfunction with the age of onset at 32 weeks of age, as scored by the beam walk test, that showed longer latencies to cross the beam and an increased number of foot slips compared with their WT littermates^21,46^. At 24 weeks of age, a significant loss of the cerebellar PCs was observed in these mice together with a progressive loss of calbindin-28k that represents a marker for neuronal dysfunction^46^. Disturbances of the electrophysiological functions have been reported in these mice in the studies on the cerebellar slices at 24 weeks of age^17,18^, in urethane-anesthetized mice also at 24 weeks of age^20^ and in awake head-fixed SCA2 mice at the age of 36 weeks^21^. The deranged calcium signaling and spine loss were also observed in SCA2-58Q mice^47^. We recently reported that SCA2-58Q mice exhibit anxiolytic behavior, decline in spatial memory, and a depressive-like state at the age of 28 weeks^16^.

Here we studied the effect of long-term i. p. injections of SK channel positive modulator CHZ on the motor, cognitive, and affective decline reported in SCA2-58Q mice^16^. It is well known that CHZ is able to cross the BBB^48,49^. Previously, CHZ treatment has rescued the motor dysfunction in murine models of EA2^27^, SCA1^24^, SCA2^21^, in mutant *CACNA1A* mice^28^, and in YAC128 HD mice^30^. Being an FDA-approved drug, CHZ has very little amount of side effects in humans^50^. However, the study of the CHZ effect on the cognitive and affective functions has not yet been carried out in ataxic mice. Interestingly, recent research on the APP/PS1 mice with Alzheimer’s disease (AD) have demonstrated that CHZ daily administration during 23 days significantly improved the MWM performance of AD mice^51^. Authors also have observed that CHZ reduced the inflammatory response in the isolated astrocytes and microglial cells treated with Abeta^51^. CHZ has also been reported as a possible alcohol-abuse treatment^52^. According to our previously published results^21^, here we also observed that injections of CHZ improved motor decline of SCA2-58Q mice in the beam walk assay (Fig. 3). CHZ injections also reduced the anxiolytic behavior observed in SCA2-58Q mice during the open field test (Fig. 4). However, treatment with CHZ did not restore the learning strategies in the ataxic mice during the MWM task (Fig. 5). Finally, CHZ ameliorated the depressive-like state in SCA2 mice during the sucrose preference test (Fig. 7). Thus, the CHZ activation of SK channels improved both ataxic and non-motor symptoms in SCA2-58Q PC-specific transgenic mice.

However, the SK activators reduce the firing rate in the cerebellar PCs^18,20^, in the noradrenergic locus coeruleus neurons^32^, and in the midbrain dopamine neurons^53^. This reduction in firing frequency can cause side effects in human patients. Here, in our loose patch experiments we also observed the significant reduction in firing frequency in both WT and SCA2 PCs (Fig. 2C). Although the precision of PC firing was significantly ameliorated in SCA2 CHZ mice (Fig. 2D, E), the CHZ-mediated reduction in firing rate required further improvement.

Folic acid (FA) is known to be involved in the DNA synthesis and repair and homocysteine recycling. FA mitigates the glutamate-induced cell death in rat hippocampal slices^37^ and in cultured mouse cerebellar granule cells^38^. FA can also protect cerebellum against homocysteine-mediated oxidative stress^54^. There is an evidence that lack of FA can provoke cognitive decline, anxiety and depression^55,56^. Thus, experiments on mice demonstrated that FA exhibits antidepressant properties in forced swimming test (FST) and tail suspension test (TST) which seem to be mediated by the serotonergic and noradrenergic systems^57^. Further studies on mice revealed that the antidepressant effect of FA might be explained as due to the normalization of the antioxidant enzymes activity and reduction of the lipid peroxidation in the hippocampus^58^. Moreover, FA ameliorated depression induced by chronic mild stress in adult rats and by maternal deprivation in pups^59^. In this research authors also demonstrated that FA significantly reduced the oxidative damage in PFC, hippocampus, amygdala, and nucleus accumbens^59^. In a dexamethasone model of depression in mice FA improved the open-field locomotor activity and TST performance as well as cerebral cortex morphology^60^. A meta-analysis has demonstrated the reduction in serum levels of folate in humans with depression when compared to individuals without depression^61^. The literature search suggests that FA supplement ameliorated the efficacy of traditional antidepressants, although FA may have antidepressant properties itself^61,62^. Long-term FA supplementation significantly reduced the serum levels of homocysteine, Aβ-42, IL-6, and TNF-α in elderly subjects with mild cognitive impairment (MCI)^63^. Moreover, FA intake also significantly improved cognitive functions of the MCI patients in the Full-Scale IQ, Information, and Digit Span tests^63^.

Therefore, the lack of FA in the organism may lead to the cognitive dysfunction and negative psychoemotional alterations. However, high dosage of FA may also exert xenobiotic activity^36^. In our patch-clump experiments we observed that 50 μM FA significantly increased the firing frequency in WT PCs, but not SCA2-58Q PCs (Fig. 2C). Interestingly, 50 μM FA significantly increased the regularity of SCA2 PCs as their firing variability (Fig. 2D) and the percentage of tonic cells (Fig. 2E) was similar to WT readings. In WT PCs the application of FA has not changed the firing variability (Fig. 2D), although it increased the number of bursting cells recorded in WT mice (Fig. 2E). The motor coordination assessment revealed that long-term i. p. injections with 30 mg/kg FA lead to longer time to traverse all the beams tested in the beam walk assay in WT mice and did not affect the latency in SCA2-58Q mice (Fig. 3D, F), although it recovered the number of foot slips on the 8 mm beam in SCA2 mice (Fig. 3G). During the open field test, FA injections did not affect the behavior of WT mice, however, FA restored the normal anxious behavior in SCA2 mice (Fig. 4B, D). During the MWM test, WT FA mice demonstrated an increase in the locomotor activity as they moved much longer distance compared to their control group (Fig. 5D). The performance of SCA2 FA group during MWM was similar to the results of WT Ctrl (Fig. 5B, D). Finally, the long-term i. p. injections with FA increased the sugar consumption in the SPT in SCA2 mice, but did not affect the behavior of WT group (Fig. 7B). Although it looks like FA improved the behavior of SCA2 mice in the open field, MWM and SPT tests, this improvement was observed due to the increase in the variability since the statistical range of the values was wide in SCA2 FA group as we can see from the box plots (Figs. 4B, D, 5D, 7B). The patch-clamp results also suggest that the usage of FA as a potential agent to facilitate symptoms in ataxic patents is not optimal as FA increased the firing frequency (Fig. 2C) and decreased the number of tonic cells (Fig. 2E) in WT mice.

The positive modulation of calcium-activated potassium channels exerted some beneficial effects in ataxic mice^18,19,21,24–30^ and in human patients^31^. Although these results seem promising, it would be desirable to improve them further. One of the possible strategies could be the combination therapy. The average dosage of the prescribed medication is limited by its side effects among other things. It is known that SK activators suppress the neuronal firing as we also observed in this study (Fig. 2C). We suppose that side effects reported in SK therapy can be due to this change in firing frequency. We demonstrated that in SCA2-58Q PCs the CHZ-mediated reduction in firing frequency was restored when FA was added to the recording solution (Fig. 2C). Thus, we may consider the CHZ-FA combination as a possible therapeutic polypill to treat ataxia.

Indeed, CHZ-FA combination recovered the motor decline in SCA2-58Q mice (Fig. 3D, F, G) without affecting the motor performance of WT mice (Fig. 3). The CHZ-FA long-term i. p. injections also reduced anxiolytic behavior of SCA2-58Q mice in the open field test (Fig. 4B, D) while WT CHZ-FA mice exhibited similar behavior to WT Ctrl group (Fig. 4). The CHZ-FA combination also improved the learning strategies in SCA2-58Q mice during the MWM test (Fig. 5B). CHZ-FA meanwhile did not affect the MWM performance of WT mice (Fig. 5). Finally, CHZ-FA ameliorated the depressive-like behavior in SCA2-58Q mice as their sucrose consumption significantly increased during the SPT trial (Fig. 5B). However, sucrose consumption was equal between WT CHZ-FA mice and WT Ctrl group (Fig. 5B).

Here we demonstrated that SK channels-mediated therapy with CHZ improved both ataxia and cognitive symptoms in SCA2-58Q mice and rescued their depressive-like behavior. However, the patch-clump experiments demonstrated that CHZ reduces the neuronal firing of cerebellar PCs. On the contrary, FA increased the firing frequency of both WT and SCA2 PCs and decreased the number of tonic cells in WT mice. FA did not affect the motor performance of SCA2 mice in the beam walk assay, although it did exert some beneficial effects in the open field, MWM, and SPT tasks. Finally, the combined CHZ-FA therapy improved motor coordination, cognitive functions, and ameliorated the mood alterations in SCA2-58Q mice. Moreover, the CHZ-FA combination did not affect the firing frequency of SCA2-58Q PCs, but significantly improved the firing precision of ataxic PCs. Thus, this CHZ-FA trial has demonstrated the ability of the combination therapy to rescue both motor and non-motor decline in ataxic mice.

## Data Availability

The datasets used and analyzed during the current study available from the corresponding author, PAE (biopolya@gmail.com), on reasonable request.
